# Design and set-up of the leptospirosis registry *LeptoScope* for epidemiology, outbreaks and clinical studies on human leptospirosis

**DOI:** 10.3389/fpubh.2025.1687249

**Published:** 2025-11-24

**Authors:** Sadrija Cukoski, Thomas Theo Brehm, Stefan Büttner, Jens Van Praet, Sebastian Dolff, Lukas Eberwein, Iker Falces-Romero, Oliver A. Cornely, Manuel Wanken, Roman-Ulrich Müller, Volker Burst, Felix C. Koehler

**Affiliations:** 1 Faculty of Medicine, Department II of Internal Medicine, Center for Molecular Medicine Cologne, University Hospital Cologne, University of Cologne, Cologne, Germany; 2 Division of Infectious Diseases, I. Department of Medicine, University Medical Center Hamburg-Eppendorf, Hamburg, Germany; 3 German Center for Infection Research (DZIF), Partner Site Hamburg-Lübeck-Borstel-Riems, Hamburg, Germany; 4 Department of Clinical Infectious Diseases, Research Center Borstel, Leibniz Lung Center, Borstel, Germany; 5 Medical Clinic III, Cardiology, Pneumology, Nephrology and Intensive Care Medicine, Klinikum Aschaffenburg-Alzenau, Aschaffenburg, Germany; 6 Department of Nephrology and Infectious Diseases, AZ Sint-Jan Brugge AV, Brugge, Belgium; 7 Department of Infectious Diseases, West German Center of Infectious Diseases, Essen University Hospital, University of Duisburg-Essen, Essen, Germany; 8 Department III of Internal Medicine, Städtisches Klinikum Solingen, Solingen, Germany; 9 Department of Microbiology and Parasitology, Hospital Universitario La Paz, IdiPAZ, CIBERINFEC, Madrid, Spain; 10 Faculty of Medicine, Department I of Internal Medicine, Excellence Center for Medical Mycology (ECMM), University Hospital Cologne, University of Cologne, Cologne, Germany; 11 Faculty of Medicine, Clinical Trials Centre Cologne (ZKS Köln), University Hospital Cologne, University of Cologne, Cologne, Germany; 12 Faculty of Medicine, Cologne Excellence Cluster on Cellular Stress Responses in Aging-Associated Diseases (CECAD), University Hospital Cologne, University of Cologne, Cologne, Germany; 13 Faculty of Medicine, Center for Rare Diseases Cologne, University Hospital Cologne, University of Cologne, Cologne, Germany; 14 Emergency Department, University of Cologne, Faculty of Medicine and University Hospital Cologne, Cologne, Germany; 15 Department of Internal Medicine (Nephrology), Einthoven Laboratory of Vascular and Regenerative Medicine, Leiden University Medical Center, Leiden, Netherlands

**Keywords:** leptospirosis, Weil’s disease, zoonosis, waterborne bacterial disease, neglected tropicaldisease, public health, outbreak, registry

## Abstract

**Objective:**

Human leptospirosis is a widespread zoonosis with endemic appearance in different parts of the world. Despite causing more than 1 million cases, nearly 60.000 deaths and 3 million disability-adjusted life-years per year, leptospirosis remains an underrecognized and neglected disease calling for multinational surveillance and international collaboration.

**Methods:**

The leptospirosis registry *LeptoScope* is a novel project enabling both international and multi-disciplinary research on *Leptospira*-caused diseases. *LeptoScope* has an electronic case report form and can be assessed on the General Data Protection Regulation compliant platform clinicalsurveys.net. Due to its modular structure, *LeptoScope* depicts or hides items according to the documented case (e.g., patients treated in outpatient setting versus patient admitted to the intensive care unit). This ensures rapid, but standardized enrolment of patients even in epidemics.

**Results:**

Information collected in *LeptoScope* include demographics, pre-existing diseases, clinical presentation and measures in addition to outcome. A multinational research team from Germany, Belgium and Spain contributed a pilot cohort of 78 cases with *Leptospira*-associated diseases to confirm *LeptoScope’s* functionality and practicality.

**Conclusion:**

*LeptoScope* is to our knowledge the first worldwide research platform on public health and clinical studies concerning *Leptospira*-associated diseases. *LeptoScope* promotes the needed collaboration at the cross-roads of public health, microbiology, infectious diseases and nephrology for an underrecognized and often neglected disease. Ensuring controlled or uncontrolled level II evidence *LeptoScope* may improve patient care and may provide evidence for robust treatment recommendations in future.

## 1 Introduction

Human leptospirosis is a widespread zoonosis caused by the spirochetes of the genus *Leptospira* (1, 2). Leptospirosis is estimated to affect more than 1 million individuals and to cause nearly 60,000 deaths per year (3). However, lacking awareness paired with challenges in diagnosis, coverage and vaccination are major challenges affecting disease burden, which is, in turn, considered underestimated (4–6). Being neglected commonly, leptospirosis is reported as a major (re-) emerging disease being endemic in many (sub-) tropical countries with a humid climate (4, 7). In industrialized countries, leptospirosis is considered an orphan disease and mostly associated to recreational or travel activities, however climate change may cause rising incidences and outbreak situations in many parts of the world (2, 8–13).

Infection is acquired through either the direct or indirect exposure to the urine of carrier animals or via contaminated soil, water or food (14). Being an infectious vasculitis, *Leptospira* enter the body through mucus membranes or small wounds in the skin as well as via conjunctivae, aspiration and swallowing. Thus, they spread via capillaries to all tissues (15). However, clinical manifestation depends on the virulence and size of the inoculum as well as the host’s immune status. Pathogenic mechanisms in leptospirosis have, therefore, to be divided into the direct damaging effect by *Leptospira* and the host immune response. The major virulence factor is *Leptospira* motility, as they disseminate rapidly from their entry to the end-organs, including the kidneys, lung, liver, eye and the central-nervous system (1).

Clinical manifestations of leptospirosis are rather unspecific, since it mimics many other diseases due to its protean clinical picture (1, 16). Notwithstanding, the most common findings of acute severe infection due to *Leptospira* are acute kidney failure (AKI), jaundice and fever (17). Besides, leptospirosis may cause less commonly pulmonary hemorrhage, myocarditis or aseptic meningitis (6). Without timely antibiotic treatment, multiple organ dysfunctions involving the kidney, liver, heart, lungs and/or central nervous system are the consequence (6, 18). This is represented by a case fatality being as high as 5 to 15% (19).

In recent years, there is a growing recognition on the long-term kidney and neurology sequelae in addition to leptospirosis associated chronic fatigue syndrome (CFS) in survivors. Especially, in endemic regions leptospirosis predispose to chronic kidney disease (CKD) and end-stage renal disease, if not treated timely, as *Leptospira* often even persist and multiply in the tubules of kidneys despite being cleaned from blood and other organs (20–22). In this context, CKD is projected to become the fifth leading cause of worldwide deaths by 2040 (23). Thus, even non-dialysis dependent CKD itself is known to be an important risk factor for cardiovascular morbidity and mortality (24).

In addition, there is a growing recognition of leptospirosis associated psychological sequelae that is not only limited to CFS but may also include depressive symptoms, anxiety and even post-traumatic stress disorders (6, 25, 26). However, despite this clinical burden that is accompanied by approximately 3 million disability-adjusted life-years (DALYs) lost per year with a productivity loss at $29.3 billion, leptospirosis remains an underrecognized neglected tropical diseases (NTD) (6, 27, 28). In this context, leading public health organizations including the World Health Organization (WHO) have yet to prioritize leptospirosis by officially adding it to their NTD lists (6, 7).

Leptospirosis is a substantial threat disproportionately affecting the poor, marginalized, and hard-to-reach population in low- and middle-income countries (7). Triggered by this immense challenge, the novel multi-center registry project *LeptoScope* was funded in March 2020. To overcome the difficulties in obtaining sufficient data in a neglected (and some parts of the world rare) and transient disease an international approach is of utmost importance aiming for a structured surveillance (29–31). *LeptoScope* promotes this urgently needed international and multi-disciplinary cooperation at the cross-roads of public health, microbiology, infectious disease and nephrology to overcome the lack of knowledge in leptospirosis.

## 2 Materials and methods

### 2.1 Study design

*LeptoScope* was established in March 2020 and is an ongoing project. As an open registry, physicians at the cross-roads of public health, microbiology, infectious diseases and nephrology are asked to contribute both epidemiological and clinical data taken from patients with leptospirosis globally in a retrospective manner. Therefore, *LeptoScope* uses an electronic case report (eCRF) programmed with the survey software EFS Leadership 7.0 Version 1.2 (Questback GmbH, Cologne, Germany) (29–31). Yielding on a simple but structured online data collection, *LeptoScope* provides a customized version of Questback’s internationally approved EFS Survey and Leadership technology (29–31). Thus, the eCRF is accessible at *www.clinicalsurveys.net* . To sum up, *LeptoScope* is an intuitional, online, high-throughput documentation platform for *Leptospira*-associated diseases. Due to its modular structure the eCRF depicts or hides items depending on the documented clinical course to ensure structured, but rapid data entry also in epidemics.

### 2.2 Data collection

Demographic data (such as age-group, sex, ethnic origin, weight, occupation, year and month of infection, the region of infection, epidemics), as well as pre-existing medical conditions, clinical signs and symptoms upon initial presentation, microbiological results and/or imaging procedures allowing diagnosis of leptospirosis are entered in the *LeptoScope* eCRF in a standardized manner (Table 1). Clinical course and therapeutic approaches including antibiotic and diuretic therapy (drug, dose, duration, administration, the reason to stop, adverse events), renal placement therapy (indication, mode of dialysis, duration, adverse events), admission to the intensive care unit (ICU) and mechanical ventilation (duration) are further collected. Outcome (i.e., overall and attributable mortality), time of hospitalization and readmission to the hospital, long-term sequelae are additionally recorded (Table 1). If available, autopsy results may be documented, too.

**Table 1:** Leptospirosis Registry—*LeptoScope.*

Category	Subcategory
Epidemiology	Age group at diagnosis, sex, weight, year and place of infection, ethnicity, occupation
Pre-existing diseases	Malignancy, HIV/AIDS, chronic kidney disease, chronic liver disease, chronic cardiovascular disease, chronic pulmonary disease, autoimmune disorder
Clinical presentation	Signs and symptoms including at admission, disease course
Diagnostics	Microbiological analyses, imaging procedures, laboratory blood and urine results
Therapeutic approach	Admission to ICU, antibiotic and diuretic treatment approaches (drug, dose, duration, administration, adverse effects), renal replacement therapy (indication, mode of dialysis, duration, adverse effects), mechanical ventilation (duration), ECMO
Outcome	Survival, time of hospitalization, readmission to the hospital, long term kidney sequelae, development of any chronic disease after leptospirosis

Information categories documented.

AIDS, acquired immunodeficiency syndrome; ECMO, extracorporeal membrane oxygenation; HIV, human immunodeficiency virus; ICU, intensive care unit.

### 2.3 Data analysis

Documented cases are directly exported in a binary format facilitating uni- and multivariate analyses in SPSS (29–31).

### 2.4 Ethics approval

*LeptoScope* is approved from the local Ethics Committee and Review Board of the University of Cologne, Germany, (Identifier of the Ethics Committee of the University of Cologne: 19–1,631) and the study is registered at clinicaltrials.gov (Identifier: NCT04288674). *LeptoScope* is conducted in accordance with the Declaration of Helsinki and the good clinical practice guidelines of the International Conference on Harmonization.

### 2.5 Data protection

All Good Epidemiological Practice Requirements (GEP) are fulfilled, as *LeptoScope* is hosted on the General Data Protection Regulation (GDPR) compliant platform clinicalsurveys.net (Questback GmbH, Cologne, Germany) that is assessed, in turn, through encrypted communication. Thus, medical confidentially is ensured at all time (32). In this context, access to collected data or the eCRF itself is limited to selected members of *LeptoScope* staff at the University Hospital Cologne, Germany. Contributors, in turn, log-in at *LeptoScope’s* eCRF via username and password and may only view and edit their own enrolled cases without entering any identifiable data, such as name or date of birth. Entered patients are automatically stored in an anonymized manner in *LeptoScope’s* database on Questback servers in Cologne, Germany. *LeptoScope’s* staff can only view these anonymized cases in the registry’s database. In case of queries, *LeptoScope’s* staff will contact the local participants to resolve these. This resolution is conducted again in retrospectively and falls under medical confidentially.

Conclusively, an informed consent is waived, since *LeptoScope* allows the anonymous collection and storage of cases, which has been independently confirmed and approved by the local Ethics Committee and Review Board of the University of Cologne, Germany, (Identifier of the Ethics Committee of the University of Cologne: 19–1631).

### 2.6 Quality control

**Figure 1:**
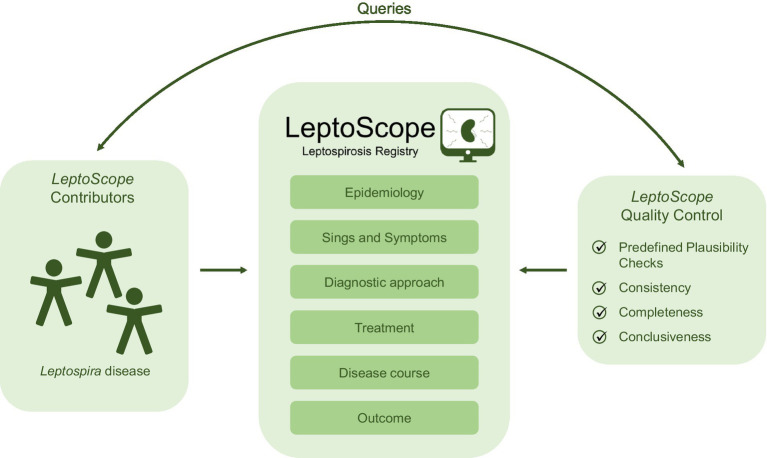
Leptospirosis registry—*LeptoScope*: data entry and quality control. Investigators enter epidemiological and clinical data of *Leptospira*-associated disease into the online electronic case report form. The *LeptoScope* quality control team checks for incorrect data entry, resolves, if necessary, relevant queries with the contributor and validates entered cases.

Automated data checks reduce the risk for false data entries. These routine quality control measures consist of both plausibility checks in addition to obligatory data input, in particular, with regard to case definitions. Besides, entered clinical cases are reviewed by a team of physicians to guarantee comprehensiveness, coherence and validity. Queries are sent to the collaborators, who, in turn, will resolve these themselves (Figure 1). Upon resolution of all queries, cases are treated as valid and used for further statistical analyses.

Moreover, extensive peer review investigations searching for incomprehensiveness and incoherence at performed both at random time points and regular interim analyses. Importantly, the anonymity of entered cases is ensured during quality control processes.

### 2.7 Funding

The Maria-Pesch-Stiftung, Cologne, Germany, supported set-up and maintenance of *LeptoScope*. Participating centres contribute voluntarily and do not receive any financial support or compensation.

## 3 Results

### 3.1 Aims and use of data

The main aims to be studied are: Delineation of the disease course due to *Leptospira* infection, recapitulation of diagnostic strategies, determination of prognostic and risk factors, analysis of antibiotic and diuretic therapeutic approaches, renal replacement therapies, mechanical ventilation and extracorporeal support systems (e.g., ECMO), in addition to the study of patient survival and, if available, long-term sequelae. Furthermore, *LeptoScope* allows for mounting leptospirosis’ incidence, even in epidemic and endemic situations. In this context, *LeptoScope* may help to identify transmission sources and the efficacy of counteractions. Besides, *LeptoScope* may improve the development or modification of clinical screening regimens, in addition to diagnostic and treatment approaches. This may benefit both patient care and the design of future guidelines.

### 3.2 Study population

*LeptoScope* is an online registry and enables the collection of leptospirosis cases on a voluntary basis at participating sites from any part in the world. Cases are eligible to be enrolled in *LeptoScope*, when the mandatory evidence of either serological (increased titer detected by microscopic agglutination test or immunofluorescence) or direct *Leptospira’s* deoxyribonucleic acid (DNA, by polymerase chain reaction (PCR)) is proved and clinical signs and symptoms of leptospirosis are apparent in parallel. Patients with a single detection of increased IgM titers, in turn, are not considered eligible due to limitation in specificity, as multiple other pathogens may cause similar clinical manifestations in the tropical setting and symptoms of leptospirosis are often unspecific.

Of note, *LeptoScope* facilitates matched patient data collection using a case–control design. This in an important pre-requisite to enable public health, in addition to socioeconomic analyses. Moreover, attributable hospitalization, morbidity and mortality may be studied. Case-controls, in turn, will be recruited in the same participating hospitals that contribute patient cases, individual matched and further stratified for gender, age, and underlying diseases. On the one hand, the healthy population (age-group- and sex-matched) will be used as case–controls for public health analyses. On the other hand, case–controls may be further matched for pre-existing disease (e.g., leptospirosis patients with CKD will be matched to a non-leptospirosis CKD patient of the same KDIGO stage, age-group and sex) in order to examine the attributable disease burden of leptospirosis (33).

Further information regarding *LeptoScope* can be found together with its sister registry – the Hantavirus Registry *HantaReg -* at www.kidneyinfection.org (29).

### 3.3 Case recruitment of a pilot-cohort in three European countries

Until March 2025, a total of 78 cases were recruited in eight centres from Germany, Belgium and Spain. These cases underwent quality control and were considered valid.

## 4 Discussion

On the one hand, leptospirosis is a neglected disease in many parts of the world. On the other hand, it is an orphan disease in industrialized countries (6, 7). This great imbalance strongly calls for international cooperation to increase disease specific knowledge and to improve the overall outcome. *LeptoScope* is the product of such an international effort at the cross-roads of public health, microbiology, infectious diseases and nephrology. *LeptoScope* simplifies international cooperation thus facilitating epidemiological in addition to public health analyses. Besides, studies of disease course including organ tropism, prognostic in addition to risk factors are aimed for. As *LeptoScope* uses a matched case-control design the study of attributable morbidity and mortality paired with the implementation of health economic analysis is feasible.

One major benefit of *LeptoScope* is its modular structure. This is an important pre-requisite for fast, but structured data entry. In recent years, serious outbreak situations of leptospirosis have occurred in different parts of the world (6, 11, 34–36). This calls for real-time monitoring of epidemics together with the performed counteractions. To face challenges in real-life monitoring during outbreaks, *LeptoScope’s* lean design, in turn, yields on easy and fast entry of patient cases.

From March 2020 until March 2025, eight centres from Germany, Belgium and Spain contributed a pilot cohort of 78 cases with *Leptospira*-associated diseases into *LeptoScope*, thus proving the registry’s functionality and practicality.

With regard to *LeptoScope’s* limitations, there are both general shortcomings of registries in addition to specific limitations applying to *LeptoScope* (29–31). Although *LeptoScope* yields on a long-time follow-up of enrolled cases, their loss may additionally hamper data quality. Furthermore, reporting and selection bias need to be considered. Data is entered in a retrospective, but anonymized manner to adhere to current data protection guidelines and to ensure protection of the privacy of enrolled patients. This results in a reduced quality of data. However, anonymous data collection and storage without the need of an informed consent is an important pre-requisite of registries to ensure everyday practicability, data enrolment together with highest levels of data protection. We have used this specific approach very successfully with large worldwide registries for different emerging infectious diseases, such as Hantavirus-associated diseases, invasive *Candida* infections and even rarer fungi in recent years (29–31).

Leptospirosis is a neglected disease and in some parts of the world an orphan disease. So, worldwide pooling cases will result in cohort analyses with sufficient cases included. This is an important pre-requisite in search for a high quality of evidence improving future design of clinical trials. Consequently, we would like to invite physicians around the world, who are dealing with leptospirosis, to participate in *LeptoScope*. This joint effort will help to better understand burdens associated with orphan and neglected infectious diseases (29–31). In this context, *LeptoScope* offers co-authorship to contributors, who meet the respective definition of the ICMJE (37). To participants not fulfilling ICMJE’s authorship criteria, a collaborator status is offered to recognize data entry (38). Of note, both author and collaborator status contribute to indices, including the h-index, which highlight a scientist’s publication performance (31, 39). For treating physicians in need for immediate reference information for challenging cases, we aim to set-up a freely available web search engine based on an extract of all valid cases in the second phase of the project.

As a conclusion, *LeptoScope* is as a novel platform allowing multi-centric collaboration to increase the knowledge in *Leptospira*-associated diseases and their burdens (6). To date, guidelines are missing and recommendations are mainly based on scarce evidence and expert opinion. In contrast, registry projects like *LeptoScope* facilitate controlled or uncontrolled level II evidence, which may guide on future guideline development and benefits in patient care (29–31, 40, 41).

## Data Availability

The original contributions presented in the study are included in the article/Supplementary material, further inquiries can be directed to the corresponding author/s.

## References

[1] Bharti AR Nally JE Ricaldi JN Matthias MA Diaz MM Lovett MA . Leptospirosis: a zoonotic disease of global importance. Lancet Infect Dis. (2003) 3:757–71. doi: 10.1016/S1473-3099(03)00830-2, PMID: 14652202

[2] Hartskeerl RA Collares-Pereira M Ellis WA. Emergence, control and re-emerging leptospirosis: dynamics of infection in the changing world. Clin Microbiol Infect. (2011) 17:494–501. doi: 10.1111/j.1469-0691.2011.03474.x, PMID: 21414083

[3] Costa F Hagan JE Calcagno J Kane M Torgerson P Martinez-Silveira MS . Global morbidity and mortality of leptospirosis: a systematic review. PLoS Negl Trop Dis. (2015) 9:e0003898. doi: 10.1371/journal.pntd.0003898, PMID: 26379143 PMC4574773

[4] Karpagam KB Ganesh B. Leptospirosis: a neglected tropical zoonotic infection of public health importance-an updated review. Eur J Clin Microbiol Infectious Diseases. (2020) 39:835–46. doi: 10.1007/s10096-019-03797-4, PMID: 31898795

[5] Herman HS Mehta S Cárdenas WB Stewart-Ibarra AM Finkelstein JL. Micronutrients and leptospirosis: a review of the current evidence. PLoS Negl Trop Dis. (2016) 10:e0004652. doi: 10.1371/journal.pntd.0004652, PMID: 27387046 PMC4936698

[6] Muñoz-Zanzi C Dreyfus A Limothai U Foley W Srisawat N Picardeau M . Leptospirosis-improving healthcare outcomes for a neglected tropical disease. Open Forum Infect Dis. (2025) 12:ofaf035. doi: 10.1093/ofid/ofaf035, PMID: 39963696 PMC11832045

[7] Tshokey T Ko AI Currie BJ Munoz-Zanzi C Goarant C Paris DH . Leptospirosis, melioidosis, and rickettsioses in the vicious circle of neglect. PLoS Negl Trop Dis. (2025) 19:e0012796. doi: 10.1371/journal.pntd.0012796, PMID: 39847571 PMC11756766

[8] van Alphen LB Lemcke Kunoe A Ceper T Kahler J Kjelso C Ethelberg S . Trends in human leptospirosis in Denmark, 1980 to 2012. Euro Surveillance. (2015) 20. doi: 10.2807/1560-7917.ES2015.20.4.21019 25655055

[9] Mori M Vane M Depoorter S Decaluwe W Vandecasteele SJ Fretin D . Outbreak of leptospirosis during a scout camp in the Luxembourg Belgian province, Belgium, summer 2012. Epidemiol Infect. (2015) 143:1761–6. doi: 10.1017/S0950268814002763 25311398 PMC9507239

[10] Brockmann S Piechotowski I Bock-Hensley O Winter C Oehme R Zimmermann S . Outbreak of leptospirosis among triathlon participants in Germany, 2006. BMC Infect Dis. (2010) 10:91. doi: 10.1186/1471-2334-10-91, PMID: 20380736 PMC2858141

[11] Poulakida I Kotsiou OS Boutlas S Stergioula D Papadamou G Gourgoulianis KI . Leptospirosis incidence post-flooding following storm Daniel: the first case series in Greece. Infect Dis Rep. (2024) 16:880–7. doi: 10.3390/idr16050069, PMID: 39311210 PMC11417790

[12] Mora C McKenzie T Gaw IM Dean JM von Hammerstein H Knudson TA . Over half of known human pathogenic diseases can be aggravated by climate change. Nat Clim Chang. (2022) 12:869–75. doi: 10.1038/s41558-022-01426-1, PMID: 35968032 PMC9362357

[13] Brehm TT Schulze Zur Wiesch J Lütgehetmann M Tappe D Eisermann P Lohse AW . Epidemiology, clinical and laboratory features of 24 consecutive cases of leptospirosis at a German infectious disease center. Infection. (2018) 46:847–53. doi: 10.1007/s15010-018-1181-x, PMID: 30019313

[14] Adler B de la Peña Moctezuma A. *Leptospira* and *Leptospirosis*. Vet Microbiol. (2010) 140:287–96. doi: 10.1016/j.vetmic.2009.03.012, PMID: 19345023

[15] Abdulkader RC Silva MV. The kidney in leptospirosis. Pediatric Nephrol. (2008) 23:2111–20. doi: 10.1007/s00467-008-0811-4, PMID: 18446381

[16] Farr RW. Leptospirosis. Clin Infect Dis. (1995) 21:1–6. 7578715 10.1093/clinids/21.1.1

[17] Yang CW. Leptospirosis renal disease: emerging culprit of chronic kidney disease unknown etiology. Nephron. (2018) 138:129–36. doi: 10.1159/000480691, PMID: 28926845

[18] Haake DA Levett PN. Leptospirosis in humans. Curr Top Microbiol Immunol. (2015) 387:65–97. doi: 10.1007/978-3-662-45059-8_5, PMID: 25388133 PMC4442676

[19] Levett PN. Leptospirosis. Clin Microbiol Rev. (2001) 14:296–326. doi: 10.1128/CMR.14.2.296-326.2001, PMID: 11292640 PMC88975

[20] Chikeka I Dumler JS. Neglected bacterial zoonoses. Clin Microbiol Infect. (2015) 21:404–15. doi: 10.1016/j.cmi.2015.04.022, PMID: 25964152 PMC4466158

[21] Yang HY Hung CC Liu SH Guo YG Chen YC Ko YC . Overlooked risk for chronic kidney disease after Leptospiral infection: a population-based survey and epidemiological cohort evidence. PLoS Negl Trop Dis. (2015) 9:e0004105. doi: 10.1371/journal.pntd.0004105, PMID: 26452161 PMC4599860

[22] Phannajit J Lertussavavivat T Limothai U Tachaboon S Avihingsanon Y Praditpornsilpa K . Long-term kidney outcomes after leptospirosis: a prospective multicentre cohort study in Thailand. Nephrology Dialysis Transplantation. (2023) 38:2182–91. doi: 10.1093/ndt/gfad030, PMID: 36746439

[23] Vanholder R Annemans L Bello AK Bikbov B Gallego D Gansevoort RT . Fighting the unbearable lightness of neglecting kidney health: the decade of the kidney. Clin Kidney J. (2021) 14:1719–30. doi: 10.1093/ckj/sfab070, PMID: 34221379 PMC8243275

[24] Tonelli M Muntner P Lloyd A Manns BJ Klarenbach S Pannu N . Risk of coronary events in people with chronic kidney disease compared with those with diabetes: a population-level cohort study. Lancet. (2012) 380:807–14. doi: 10.1016/S0140-6736(12)60572-8, PMID: 22717317

[25] Goris MG Kikken V Straetemans M Alba S Goeijenbier M van Gorp EC . Towards the burden of human leptospirosis: duration of acute illness and occurrence of post-leptospirosis symptoms of patients in the Netherlands. PLoS One. (2013) 8:e76549. doi: 10.1371/journal.pone.0076549, PMID: 24098528 PMC3789694

[26] Prinsen G Baker M Benschop J Collins-Emerson J Douwes J Fayaz A . "We don't really do doctors." Messages from people diagnosed with occupational leptospirosis for medical professionals on infection, hospitalisation, and long-term effects. Heliyon. (2023) 9:e19303. doi: 10.1016/j.heliyon.2023.e19303, PMID: 37674827 PMC10477488

[27] Torgerson PR Hagan JE Costa F Calcagno J Kane M Martinez-Silveira MS . Global burden of leptospirosis: estimated in terms of disability adjusted life years. PLoS Negl Trop Dis. (2015) 9:e0004122. doi: 10.1371/journal.pntd.0004122, PMID: 26431366 PMC4591975

[28] Agampodi S Gunarathna S Lee JS Excler JL. Global, regional, and country-level cost of leptospirosis due to loss of productivity in humans. PLoS Negl Trop Dis. (2023) 17:e0011291. doi: 10.1371/journal.pntd.0011291, PMID: 37616329 PMC10482283

[29] Koehler FC Blomberg L Brehm TT Büttner S Cornely OA Degen O . Development and design of the hantavirus registry - HantaReg - for epidemiological studies, outbreaks and clinical studies on hantavirus disease. Clin Kidney J. (2021) 14:2365–70. doi: 10.1093/ckj/sfab053, PMID: 34754431 PMC8573013

[30] Koehler P Arendrup MC Arikan-Akdagli S Bassetti M Bretagne S Klingspor L . ECMM CandiReg-A ready to use platform for outbreaks and epidemiological studies. Mycoses. (2019) 62:920–7. doi: 10.1111/myc.12963, PMID: 31271702 PMC7614793

[31] Seidel D Durán Graeff LA Vehreschild M Wisplinghoff H Ziegler M Vehreschild JJ . FungiScope(™) -global emerging fungal infection registry. Mycoses. (2017) 60:508–16. 28730644 10.1111/myc.12631

[32] Hoffmann W Latza U Baumeister SE Brunger M Buttmann-Schweiger N Hardt J . Guidelines and recommendations for ensuring good epidemiological practice (GEP): a guideline developed by the German Society for Epidemiology. Eur J Epidemiol. (2019) 34:301–17. doi: 10.1007/s10654-019-00500-x, PMID: 30830562 PMC6447506

[33] KDIGO. Clinical practice guideline for the evaluation and Management of Chronic Kidney Disease. Kidney Int. (2024) 2024, 105:S117–s314. 10.1016/j.kint.2023.10.018 38490803

[34] Dreesman J Toikkanen S Runge M Hamschmidt L Lüsse B Freise JF . Investigation and response to a large outbreak of leptospirosis in field workers in Lower Saxony, Germany. Zoonoses Public Health. (2023) 70:315–26. doi: 10.1111/zph.13027, PMID: 36692076

[35] Waranius B Tillman C Van Houten C Harrist A Digianantonio R Hasel H . Human case of leptospirosis during a canine disease outbreak - Wyoming, 2023. MMWR Morb Mortal Wkly Rep. (2024) 73:602–6. doi: 10.15585/mmwr.mm7327a1, PMID: 38990767 PMC11254349

[36] Caraballo L Rangel Y Reyna-Bello A Muñoz M Figueroa-Espinosa R Sanz-Rodriguez CE . Outbreak of intermediate species Leptospira venezuelensis spread by rodents to cows and humans in *L. interrogans*-endemic region, Venezuela. Emerg Infect Dis. (2024) 30:1514–22. doi: 10.3201/eid3008.231562, PMID: 39043385 PMC11286060

[37] International Committee of Medical Journal Editors. (2025). Available online at: https://icmje.org/recommendations/browse/10.12771/emj.2024.e48 PMC12093628 40704003

[38] Kalager M Adami HO Bretthauer M. Recognizing Data Generation. N Engl J Med. (2016) 374:1898. doi: 10.1056/NEJMc1603789, PMID: 27096437

[39] Hirsch JE. An index to quantify an individual's scientific research output. Proc Natl Acad Sci USA. (2005) 102:16569–72. doi: 10.1073/pnas.0507655102, PMID: 16275915 PMC1283832

[40] Evidence-Based Medicine. A new approach to teaching the practice of medicine. JAMA. (1992) 268:2420–5. doi: 10.1001/jama.1992.03490170092032, PMID: 1404801

[41] Wilson MC Hayward RS Tunis SR Bass EB Guyatt G. Users' guides to the medical literature. VIII. How to use clinical practice guidelines. B. What are the recommendations and will they help you in caring for your patients? The evidence-based medicine working group. JAMA. (1995) 274:1630–2. doi: 10.1001/jama.1995.03530200066040, PMID: 7474251

