# Supplementary material for: Design and set-up of the leptospirosis registry LeptoScope for epidemiology, outbreaks and clinical studies on human leptospirosis
Source: Front Public Health. 2025 Nov 24;13:1687249. doi: 10.3389/fpubh.2025.1687249 (PMC12682807; doi:10.3389/fpubh.2025.1687249)
Supplement: Supplementary file 1 [file Data_Sheet_1.PDF]

UNIKLINIK KÖLN
Klinik II für Innere Medizin
Nephrologie, Rheumatologie, Diabetologie
und Allgemeine Innere Medizin

Electronic Case Report Form

LeptoScope

Leptospirosis Registry

Felix C. Koehler, MD

Volker Burst, MD

Version 1.0

November 26, 2019

Kerpener Straße 62
50937 Köln
Telefon +49 221 478-0
Telefax +49 221 478-4095

www.uk-koeln.de

Universitätsklinikum Köln (AöR)
Vorstand: Prof. Dr. Edgar Schömig (Vorsitzender und Ärztlicher Direktor) • Dipl.-Kfm. Günter Zwilling (Kaufmännischer Direktor)
Prof. Dr. Thomas Krieg (Dekan) • Vera Lux (Pflegedirektorin) • Prof. Dr. Peer Eysel (stellv. Ärztlicher Direktor)
Bank für Sozialwirtschaft Köln • BLZ: 370 205 00 • Konto: 815 0000 • IBAN: DE04 3702 0500 0008 1500 00 • BIC: BFSWDE33XXX
Steuernummer: 223/5911/1092 • Ust-IdNr.: DE 215 420 431 • IK: 260 530 283
ÖPNV: Straßenbahn Linie 9 Hst. Lindenburg, Linie 13 Hst. Gleueler Str./Gürtel • Bus Linie 146 Hst. Leiblplatz

LeptoScope

Leptospirosis Registry

Go to: -- Choose Page--

General Setup

You are documenting the following patient:
ID: 30e8a473-211b-4dee-a346-2420b82c9047

⇒ Please read the box below carefully before starting the questionnaire.

DEFINE THE DAY OF DIAGNOSIS - Day Zero

In the following questionnaire, you must often refer to the day of diagnosis of invasive Leptospirosis disease (ILD).
The day of diagnosis is considered the day that the first positive microbiological (e.g. serology or PCR) or histological test result was provided to the treating physician triggering systemic treatment.

In the case of post mortem diagnosis, the day of death is considered to be the day of diagnosis.

Inclusion and exclusion criteria

For help please contact Felix Köhler (felix.koehler@uk-koeln.de)

There was microbiological or histological evidence of Leptospirosis infection with consistent signs of invasive Leptospirosis disease	○ Yes	○ No
There was positive microbiological or histological evidence of Leptospirosis infection without signs of invasive Leptospirosis disease	○ Yes	○ No
Case Control Patient	○ Yes	○ No

Country from which this case is documented
Country name in English

-- please select --

If you document this case on behalf of your PI or the treating physician, please indicate her or his name.

Institution from where this case is being documented
Institute, Department, City

Please classify your institution according to the level of care you provide.

- ○ Primary care (e.g. general practitioner)
- ○ Secondary care (e.g. medical specialist)
- ○ Tertiary care (e.g. University Hospital, Reference Center)
- ○ Outpatient clinic
- ○ Other. Please specify:

For help please contact us.

Continue

LeptoScope
Leptospirosis Registry

Go to: -- Choose Page--

Details on the Leptospirosis species causing invasive Leptospirosis disease

Species causing invasive Leptospirosis disease.
In case of case control patient, please select not applicable.

Was more than one causative pathogen identified?
- ◯ No
- ◯ Yes. Please specify further pathogens:

If Leptospirosis infection was imported from another country, please state from which country.
If Leptospirosis infection was not imported, please select "No" from the top of the list.
-- please select --

Outbreak situation
Did invasive Leptospirosis disease occurred during an outbreak situation.
- ☐ Yes
- ☐ No
- ☐ Unknown

Documentation of this case in any other registries?
- ◯ No
- ◯ Yes. Please specify:

Case already published?
- ◯ No
- ◯ Yes. Please specify (digital object identifier - DOI):

Exposure area
Please provide information regarding patient's exposure to Leptospirosis
- ☐ Rural area
- ☐ Urban area
- ☐ Suburban area
- ☐ Unknown

Occupation at the time of diagnosis of invasive Leptospirosis disease?
- ◯ Agriculture / ranching
- ◯ Construction / landscaping
- ◯ Forestry / parks / outdoor recreation
- ◯ Cleaning
- ◯ Oil field
- ◯ Scientific animal work
- ◯ Unemployed
- ◯ Unknown

Assumed exposure?
- ◯ Home
- ◯ Occupational
- ◯ Recreational
- ◯ Other
- ◯ Unknown

For help please contact us.

Back | Continue

LeptoScope

Leptospirosis Registry

Go to: -- Choose Page--

Patient Setup

Sex

◯ Female ◯ Male

Weight

[ ] kg

Year of invasive leptospirosis disease

-- please select --

Ethnic origin

If the ethnic origin is unclear, please select *Unknown*.

-- please select --

To which age group did the patient belong at the time of diagnosis of invasive leptospirosis disease?

Child/Adolescent:

◯ Preterm

◯ Neonate (< 1 month)

◯ Infant (1 - 12 months)

◯ 1 - 6 years

◯ 7 - 11 years

◯ 12 - 17 years

Adult:

◯ 18 - 29 years

◯ 30 - 49 years

◯ 50 - 69 years

◯ 70 - 89 years

◯ ≥ 90 years

Was the patient vaccinated against leptospirosis before invasive leptospirosis disease?

◯ No

◯ Yes. Please specify vaccination: [ ]

◯ Unknown

For help please contact us.

Back | Continue

LeptoScope

Leptospirosis Registry

Go to: -- Choose Page--

Pre-existing diseases

Which pre-existing disease were present prior diagnosis of invasive leptospirosis disease?

- [ ] Hematological/Oncological disease
- [ ] HIV/AIDS
- [ ] Solid organ transplantation
- [ ] Chronic cardiovascular disease
- [ ] Chronic liver disease
- [ ] Chronic pulmonary disease
- [ ] Chronic renal disease
- [ ] Alcoholism
- [ ] Diabetes mellitus
- [ ] IV drug abuse
- [ ] Rheumatic diseases/Autoimmune disorder
- [ ] Obesity (BMI >30) or Underweight (BMI <18.5), please indicate BMI:
- [ ] Other underlying disease not mentioned above (e.g. neurological disorder)
- [ ] **No pre-existing disease**

For help please contact us.

Back Continue

LeptoScope
Leptospirosis Registry

Go to: -- Choose Page--

Hematological/Oncological disorder

You stated the patient was diagnosed with a hematological/oncological disorder prior to diagnosis of invasive Leptospirosis disease. Please provide further details on the condition of the patient.

Type of disease

- ◯ Acute Leukemia
- ◯ Aplastic Anemia
- ◯ Chronic Leukemia
- ◯ Lymphoma
- ◯ Multiple Myeloma
- ◯ Myelodysplastic Syndrome
- ◯ Solid Tumor
- ◯ Other

Details on the diagnosis

State of disease prior to diagnosis of the invasive Leptospirosis disease

- ◯ de novo (first line)
- ◯ First relapse
- ◯ Second or later relapse
- ◯ Unknown

For help please contact us.

Back | Continue

LeptoScope

Leptospirosis Registry

Go to: -- Choose Page--

Solid Organ Transplantation

You stated the patient received a solid organ transplantation (SOT) before invasive Leptospirosis disease. Please provide further details.

Organ(s) transplanted

- [ ] Heart
- [ ] Intestine
- [ ] Kidney
- [ ] Liver
- [ ] Lung
- [ ] Pancreas
- [ ] Other. Please specify: ______

Time span between SOT and diagnosis of invasive Leptospirosis disease

______ months

For help please contact us.

Back | Continue

LeptoScope

Leptospirosis Registry

Go to: -- Choose Page--

HIV/AIDS

You stated the patient was diagnosed with HIV/AIDS prior to diagnosis of invasive Leptospirosis disease. Please provide further details.

Most recent CD4-cell count prior to diagnosis of invasive Leptospirosis disease.

- ◯ CD4 cell count in cells/µL: ______
- ◯ Unknown

Most recent viral load prior to diagnosis of invasive Leptospirosis disease

- ◯ Viral load in copies/mL: ______
- ◯ Below level of detection
- ◯ Unknown

Was the patient receiving Antiretroviral therapy (ART) prior to diagnosis of invasive Leptospirosis disease?

- ◯ Yes
- ◯ No
- ◯ Unknown

For help please contact us.

Back | Continue

LeptoScope

Leptospirosis Registry

Go to: -- Choose Page--

Chronic liver disease

You stated the patient suffered from chronic liver disease prior to diagnosis of invasive Leptospirosis disease. Please provide further details on the disease at the time of diagnosis of invasive Leptospirosis disease.

Etiology
Multiple choices possible

- ☐ Chronic liver disease due to Hepatitis B
- ☐ Chronic liver disease due to Hepatitis C
- ☐ Alcoholic liver disease
- ☐ Non-alcoholic fatty liver disease
- ☐ Drug induced liver disease. Please provide details: ________
- ☐ Hemochromatosis
- ☐ Wilson's disease
- ☐ Primary biliary cholangitis
- ☐ Primary sclerosing cholangitis
- ☐ Other. Please provide details: ________

Duration prior to diagnosis of invasive Leptospirosis disease

____ month

Severity according to Child-Pugh Classification

- ○ Child A
- ○ Child B
- ○ Child C
- ○ Unknown

Severity according to Model for End-Stage Liver Disease (MELD) score (Calculator)

- ○ MELD score: ____
- ○ Unknown

For help please contact us.

Back Continue

LeptoScope
Leptospirosis Registry

Go to: -- Choose Page--

Chronic cardiovascular disease

You stated the patient suffered from chronic cardiovascular disease prior to diagnosis of invasive Leptospirosis disease. Please provide further details on the disease at the time of diagnosis of invasive Leptospirosis disease.

Etiology
Multiple choices possible

- ☐ Hypertension
- ☐ Congestive heart failure
- ☐ Coronary heart disease
- ☐ Heart arrhythmia
- ☐ Valvular heart disease
- ☐ Other. Please provide details: ______

Duration prior to diagnosis of invasive Leptospirosis disease

____ month

Severity of arterial hypertension according to the European Society of Hypertension

- ◯ Grade 1 hypertension (systolic 140-159 mmHg and/or diastolic 90-99 mmHg)
- ◯ Grade 2 hypertension (systolic 160-179 mmHg and/or diastolic 100-109 mmHg)
- ◯ Grade 3 hypertension (systolic >180 and/or diastolic >110 mmHg)
- ◯ Unknown
- ◯ Not applicable

In case of congestive heart failure, please specify severity of congestive heart failure according to the Ney York Heart Association (NYHA) classification at the time of diagnosis of invasive Leptospirosis disease.

- ◯ NYHA class 1 (No limitation of physical activity)
- ◯ NYHA class 2 (slight limitation of physical activity)
- ◯ NYHA class 3 (moderate limitation of physical activity)
- ◯ NYHA class 4 (Unable to carry on any physical activity without discomfort)
- ◯ Unknown
- ◯ Not applicable

For help please contact us.

Back | Continue

LeptoScope
Leptospirosis Registry

Go to: -- Choose Page--

Chronic renal disease

You stated the patient suffered from chronic renal disease prior to diagnosis of invasive Leptospirosis disease. Please provide further details on the disease at the time of diagnosis of invasive Leptospirosis disease.

Etiology
Multiple choices possible

- ☐ Vascular kidney disease. Please specify: ____
- ☐ Primary glomerular disease. Please specify: ____
- ☐ Secondary glomerular disease. Please specify: ____
- ☐ Congenital disease. Please specify: ____
- ☐ Tubulointestinal disease. Please specify ____
- ☐ Obstructive nephropathy. Please specify: ____
- ☐ Idiopathic kidney disease. Please specify: ____
- ☐ Other. Please specify: ____

Duration prior to diagnosis of invasive Leptospirosis disease

____ month

Current stage

- ◯ Stage I (GFR > 90)
- ◯ Stage II (GFR 60-89)
- ◯ Stage III (GFR 30-59)
- ◯ Stage IV (GFR 15-29)
- ◯ Stage V (GFR < 15 or dialysis)
- ◯ Unknown

Albuminuria

- ◯ None
- ◯ Microalbuminuria (albumin creatinine ratio 30-300 µg/mg)
- ◯ Macroalbuminuria (albumin creatinine ratio > 300 µg/mg)
- ◯ Unknown

Did the patient undergo dialysis prior invasive Leptospirosis disease?

- ◯ Hemodialysis
- ◯ Peritoneal dialysis
- ◯ No dialysis
- ◯ Unknown

For help please contact us.

Back | Continue

LeptoScope
Leptospirosis Registry

Go to: -- Choose Page--

Chronic pulmonary disease

You stated the patient suffered from chronic pulmonary disease prior invasive Leptospirosis disease. Please provide further details.

Type of chronic pulmonary disease
Multiple choices possible

- ☐ Asthma
- ☐ Chronic obstructive pulmonary disease (COPD)
- ☐ Cystic fibrosis
- ☐ Idiopathic pulmonary fibrosis
- ☐ Other. Please specify: ______

Duration prior to diagnosis of invasive Leptospirosis disease

____ month

Most recent vital capacity <u>prior</u> to diagnosis of invasive Leptospirosis disease

- ◯ Vital capacity (L.) ____
- ◯ Unknown

Most recent forced expiratory volume in one second (FEV1) <u>prior</u> to diagnosis of invasive Leptospirosis disease

- ◯ forced expiratory volume in one second (FEV1) (% of the age-appropriate standard value) ____
- ◯ Unknown

For help please contact us.

Back | Continue

LeptoScope

Leptospirosis Registry

Go to: -- Choose Page--

Diabetes mellitus

You stated the patient suffered from Diabetes mellitus prior invasive Leptospirosis disease. Please provide further details.

Duration prior to diagnosis of invasive Leptospirosis disease.

[ ] month

Insulin dependent at time of diagnosis of the invasive Leptospirosis disease?

- ○ Yes
- ○ No
- ○ Unknown

End-organ damage present?
Multiple choices possible

- ☐ No
- ☐ Coronary artery disease
- ☐ Diabetic foot ulcers
- ☐ Nephropathy
- ☐ Polyneuropathy
- ☐ Retinopathy
- ☐ Stroke
- ☐ Other. Please provide details: [ ]

Most recent Hb1AC prior to diagnosis of invasive Leptospirosis disease

[ ] %

For help please contact us.

Back | Continue

LeptoScope

Leptospirosis Registry

Go to: -- Choose Page--

Rheumatic/Autoimmune disease

You stated the patient was diagnosed with a rheumatic/autoimmune disease prior to diagnosis of invasive Leptospirosis disease. Please provide further details.

Etiology
Multiple choices possible

- ☐ Rheumatoid arthritis
- ☐ Systemic Lupus Erythematosus
- ☐ Sjörgen's syndrome
- ☐ Systemic fasciitis
- ☐ Eosinophilic fasciitis
- ☐ Polymyositis and dermatomyositis
- ☐ Mixed connective tissue disease
- ☐ Relapsing polychondritis
- ☐ Other. Please provide details: ____________

Time span between onset of rheumatic/autoimmune disease and diagnosis of invasive Leptospirosis disease

____ month

State of disease prior to diagnosis of invasive Leptospirosis disease

- ○ Acute attack
- ○ Chronic active
- ○ Remission
- ○ Not applicable
- ○ Unknown

For help please contact us.

Back | Continue

LeptoScope
Leptospirosis Registry

Go to: -- Choose Page--

Clinical Signs and Symptoms

Please state the clinical signs and symptoms attributed to invasive Leptospirosis disease at day of diagnosis.

	Yes	No	Unknown
Fever	○	○	○
Oliguria (< 500mL urine/ day)	○	○	○
Anuria (< 100mL urine/ day)	○	○	○
Edema	○	○	○
Uremic fetor	○	○	○
Neurological disorder	○	○	○
Blurred vision	○	○	○
Tachycardia	○	○	○
Hypotension	○	○	○
Shock	○	○	○
Cough	○	○	○
Dyspnea	○	○	○
Hemoptysis	○	○	○
Dehydration	○	○	○
Diarrhea	○	○	○
Anorexia	○	○	○
Petechiae / Hemorrhage	○	○	○
Rash	○	○	○
Jaundice	○	○	○
Hepatomegaly	○	○	○
Conjunctival suffusion	○	○	○
Pain. Please specify: ______	○	○	○
Other. Please provide details: ______	○	○	○

For help please contact us.

Back Continue

LeptoScope
Leptospirosis Registry

Go to: -- Choose Page--

Vital signs

Please provide vital signs at day of diagnosis of the invasive Leptospirosis disease, after 24h, after 72h and at the day of discharge.

Day = Select day of procedure relative to the **day of diagnosis of the invasive Leptospirosis disease**, e.g. 0 (day of diagnosis) or -5 (day 5 before diagnosis) or 7 (day 7 after diagnosis).

DEFINE THE DAY OF DIAGNOSIS - Day Zero
The day of diagnosis is considered the day that the first positive microbiological (e.g. serology or PCR) or histological test result was provided to the treating physician triggering systemic treatment.

In the case of post mortem diagnosis, the day of death is considered to be the day of diagnosis.

	**Heart rate**	**Systolic blood pressuresite**	**Diastolic blood pressure**	**Temperature**	**Breathing rate**	**Oxygen saturation**
example:	120mmHg	95mmHg	40mmHg	38,9°C	21/min	92%
d0.	/min	mmHg	mmHg	°C	/min	%
24h.	/min	mmHg	mmHg	°C	/min	%
72h.	/min	mmHg	mmHg	°C	/min	%
discharge.	/min	mmHg	mmHg	°C	/min	%

For help please contact us.

Back Continue

LeptoScope

Leptospirosis Registry

Go to: -- Choose Page--

Leptospirosis Evidence

Please specify microbiological and histological efforts undertaken to diagnose invasive Leptospirosis disease (ILD). Follow-up analyses not used to establish diagnosis of the invasive Leptospirosis disease do not need to be documented.

Day = Select day of procedure relative to the **day of diagnosis of the invasive Leptospirosis disease**, e.g. 0 (day of diagnosis) or -5 (day 5 before diagnosis) or 7 (day 7 after diagnosis).

DEFINE THE DAY OF DIAGNOSIS - Day Zero
The day of diagnosis is considered the day that the first positive microbiological (e.g. serology or PCR) or histological test result was provided to the treating physician triggering systemic treatment.

In the case of post mortem diagnosis, the day of death is considered to be the day of diagnosis.

	Procedure	sample site	Detection of ILD	Day	Description of findings	Leptospira spp. identified (species)
example:	Immuno assay (ELISA)	Blood	yes	0	positive immuno assay	Leptospira interogans
1.						
2.						
3.						
4.						
5.						
6.						
7.						
8.						

For help please contact us.

Back | Continue

LeptoScope
Leptospirosis Registry

Go to: -- Choose Page--

Imaging Procedures

Please provide further details on imaging procedures performed to diagnose invasive Leptospirosis disease (ILD). Follow-up imaging procedures not used to establish the diagnosis of invasive Leptospirosis disease or evaluate treatment response do not need to be documented.

Day = Select day of procedure relative to the day of diagnosis of invasive Leptospirosis disease, e.g. 0 (day of diagnosis) or -5 (day 5 before diagnosis) or 7 (day 7 after diagnosis).

Day of Diagnosis - Day Zero

The day of diagnosis is considered the day that the first positive microbiological (e.g. serology or PCR) or histological test result was provided to the treating physician triggering systemic treatment.
In the case of post mortem diagnosis, the day of death is considered to be the day of diagnosis.

	Procedure	Region	Contrast	Signs of ILD	Day	Details
example:	CT	Chest	enhanced	yes	-2	Bilateral lower lobe infiltrates
1.						
2.						
3.						
4.						
5.						
6.						
7.						
8.						
Other.						

If you wish to provide further information on imaging procedures, please use the space provided below.

☐ Please check box if **no imaging procedures were performed for diagnosis of invasive Leptospirosis disease.**

For help please contact us.
Back Continue

LeptoScope

Leptospirosis Registry

Go to: -- Choose Page--

Disease course of invasive Leptospirosis disease

Which of the following were present during the disease course of invasive Leptospirosis syndrome

- [ ] Acute kidney injury
- [ ] Hemorrhage
- [ ] Acute liver failure
- [ ] Acute respiratory distress syndrome
- [ ] Acute perimyocarditis / Arrhythmia
- [ ] Acute neurological disorder
- [ ] Ocular manifestation
- [ ] Other. Please specify: ____________

Was the patient admitted to ICU during course of invasive Leptospirosis disease?

- [ ] Yes
- [ ] No

For help please contact us.

Back Continue

LeptoScope

Leptospirosis Registry

Go to: -- Choose Page--

Antibiotic therapy

Please provide details on antibiotic therapy.

Please specify **Start and Stop day** of each antibiotic treatment relative to the day of diagnosis of invasive Leptospirosis disease, e.g. 0 (day of diagnosis) or 7 (day 7 after diagnosis).

DEFINE THE DAY OF DIAGNOSIS - Day Zero

In the following questionnaire, you must often refer to the day of diagnosis of invasive Leptospirosis disease (ILD).
The day of diagnosis is considered the day that the first positive microbiological (e.g. serology or PCR) or histological test result was provided to the treating physician triggering systemic treatment.

In the case of post mortem diagnosis, the day of death is considered to be the day of diagnosis.

If **Drug-related adverse event(s) (AE)** occurred, please provide further details.

	Drug	Start Day	Stop Day	Dosage [mg]; [IU]	Frequency		Adminis-tration	Reason for Stop	Comments e.g. AEs, other drug
**example:**	Cefotaxime	0	5	1000	4x	/ Day	iv	Completed treatment	
1.						/			
2.						/			
3.						/			
4.						/			
5.						/			

☐ Please check box if **no antibiotic therapy was administered** .

For help please contact us.

Back | Continue

LeptoScope
Leptospirosis Registry

Go to: -- Choose Page--

Treatment in the Intensive Care Unit (ICU)

You stated the patient was treated in ICU. Please provide further details.

Reason(s) for admission to the ICU

- [ ] Medical
- [ ] Surgical
- [ ] Neurological
- [ ] Neurosurgical
- [ ] Pediatric
- [ ] Other
- [ ] Unknown

Which of the following were present during ICU stay?

- [ ] Central venous catheter
- [ ] Shaldon-Catheter
- [ ] Renal replacement therapy
- [ ] Mechanical ventilation
- [ ] Extracorporeal membrane oxygenation (ECMO)
- [ ] Inotropic support
- [ ] Intra aortic balloon pump (IABP)
- [ ] Parenteral nutrition
- [ ] Other
- [ ] Unknown

Which of the following were present during ICU stay?
Condition:

- [ ] Acute respiratory distress syndrome (ARDS)
- [ ] Cardiac arrhythmia
- [ ] Circulatory insufficiency
- [ ] Liver failure
- [ ] Major bleeding
- [ ] Renal failure
- [ ] Sepsis
- [ ] Other

Begin of mechanical ventilation (in relation to day 0)
e.g. day -1
day

End of mechanical ventilation (in relation to day 0)
e.g. day 14
day

Duration of whole ICU stay
day

For help please contact us.

Back | Continue

Go to: -- Choose Page--

Acute kidney injury

You stated the patient suffered from acute kidney injury during disease course of invasive Leptospirosis disease. Please provide further details concerning acute kidney injury.

Which stage of acute kidney injury according to the Kidney Improving Global Outcome (KDIGO) criteria?

KDIGO stage 1: Rise or serumcreatinine > 26.5 µmol/L (0.3 mg/dL) in 48 hours or > 1.5-2.0 fold increase in 7 days. Urine production < 0.5 mL / kg body weight over 6-12 hours.

KDIGO stage 2: Rise or serumcreatinine > 2.0-2.9 fold increase in 7 days. Urine production < 0.5 mL / kg body weight > 12 hours.

KDIGO stage 3: Rise or serumcreatinine > 353.6 µmol/L (4.0mg/dL) or > 3 fold increase in 7 days or start of renal replacement therapy (in case of patients < 18 years of age: decrease of the eGFR below 35mL/min/1.73m. Urine production < 0.3 mL / kg body weight > 24h or anuria > 12 h.

- ◯ Stage 1
- ◯ Stage 2
- ◯ Stage 3

Did the patient develop proteinuria during acute kidney injury?
protein creatinine ratio (mg/g)

[ ] mg/g

Did the patient devolop hematuria during invasive Leptospirosis disease?

- ◯ Macrohematuria
- ◯ Microhematurie
- ◯ None
- ◯ Not done

For help please contact us.

Back Continue

LeptoScope

Leptospirosis Registry

Go to: -- Choose Page--

Acute kidney injury

Please provide details on the laboratory findings during acute kidney injury.

Please state blood urea nitrogen (BUN), creatinine and estimated glomerular filtration rate (eGFR) at day of diagnosis, as well as during course of invasive Leptospirosis disease.

DEFINE THE DAY OF DIAGNOSIS - Day Zero

In the following questionnaire, you must often refer to the day of diagnosis of invasive Leptospirosis disease (ILD).

The day of diagnosis is considered the day that the first positive microbiological (e.g. serology or PCR) or histological test result was provided to the treating physician triggering systemic treatment.

In the case of post mortem diagnosis, the day of death is considered to be the day of diagnosis.

	Please select	BUN/Urea	Creatinine
day 0.		mg/dL	mg/dL
day 1.		mg/dL	mg/dL
day 2.		mg/dL	mg/dL
day 3.		mg/dL	mg/dL
day 4.		mg/dL	mg/dL
day 5.		mg/dL	mg/dL
day 6.		mg/dL	mg/dL
day 7.		mg/dL	mg/dL
week 2.		mg/dL	mg/dL
week 3.		mg/dL	mg/dL
week 4.		mg/dL	mg/dL

For help please contact us.

Back Continue

LeptoScope
Leptospirosis Registry

Go to: -- Choose Page--

Diuretic therapy

Please provide details on diuretic therapy.

Please specify **Start and Stop day** of each diuretic treatment relative to the day of diagnosis of invasive Leptospirosis disease, e.g. 0 (day of diagnosis) or 7 (day 7 after diagnosis).

DEFINE THE DAY OF DIAGNOSIS - Day Zero

In the following questionnaire, you must often refer to the day of diagnosis of invasive Leptospirosis disease (ILD).

The day of diagnosis is considered the day that the first positive microbiological (e.g. serology or PCR) or histological test result was provided to the treating physician triggering systemic treatment.

In the case of post mortem diagnosis, the day of death is considered to be the day of diagnosis.

If **Drug-related adverse event(s) (AE)** occurred, please provide further details.

	Diuretic drug	Start Day	Stop Day	Dosage [mg]	Frequency			Adminis-tration	Reason for Stop	Comments e.g. AEs, other drug
example:	Furosemide	-1	0	40	2x	/	Day	iv	failure	Visual disturbances
1.						/				
2.						/				
3.						/				
4.						/				
5.						/				
6.						/				
7.						/				
8.						/				

☐ Please check box if **no diuretic therapy was administered** .

For help please contact us.

Back | Continue

LeptoScope
Leptospirosis Registry

Go to: -- Choose Page--

Renal replacement therapy

Please provide details on renal replacement therapy.

Please specify **Start and Stop day** of each renal replacement treatment relative to the day of diagnosis of invasive Leptospirosis disease, e.g. 0 (day of diagnosis) or 7 (day 7 after diagnosis).

DEFINE THE DAY OF DIAGNOSIS - Day Zero

In the following questionnaire, you must often refer to the day of diagnosis of invasive Leptospirosis disease.

The day of diagnosis is considered the day that the first positive microbiological (e.g. serology or PCR) or histological test result was provided to the treating physician triggering systemic treatment.

In the case of post mortem diagnosis, the day of death is considered to be the day of diagnosis.

If **renal replacement therapy related adverse event(s) (AE)** occurred, please provide further details.

	Indication for dialysis	Mode of dialysis	Start Day	Stop Day	Frequency per week	Reason for Stop	Comments e.g. AEs, complication
example:	Hypervolemia	Sustained low efficiency dialysis (SLED)	0	7	2x	Completed treatment	Upper gastrointestinal bleeding
1.							
2.							
3.							
4.							
5.							

☐ Please check box if **no dialysis was performed** .

For help please contact us.

Back Continue

LeptoScope
Leptospirosis Registry

Go to: -- Choose Page--

Hemorrhage

You stated the patient suffered from hemorrhage during disease course of invasive Leptospirosis disease. Please provide details concerning hemorrhage.

Duration of thrombocytopenia (< 50000 platelets/µl)

[ ] days

Did the patient receive platelet or erythrocyte concentrations during disease course of ILD?

- ◯ Yes
- ◯ No

Major bleeding event during thrombocytopenia?
Major bleeding event is defined by the need of transfusion of erythrocyte concentrates or by the need of endoscopic or surgical interventions to stop bleeding (e.g. coloscopy in case of lower gastrointestinal bleeding)

- ◯ Intracranial bleeding
- ◯ Intracerebral bleeding
- ◯ Thoracic bleeding
- ◯ Abdominal bleeding
- ◯ Gastrointestinal bleeding
- ◯ Urogenital bleeding
- ◯ Other. Please specify: [ ]
- ◯ No

☐ Please check box if **no thrombocytopenia or hemorrhage was present.**

For help please contact us.

Back | Continue

LeptoScope
Leptospirosis Registry

Go to: -- Choose Page--

Acute liver failure

Please provide details on acute liver failure.

Please state serum bilirubin and transaminase levels (AST = aspartate aminotransferase, ALT = alanine aminotransferase) at day of diagnosis, as well as at day 7, 14, 21, 28, 35 and 42 after diagnosis.

DEFINE THE DAY OF DIAGNOSIS - Day Zero

In the following questionnaire, you must often refer to the day of diagnosis of invasive Leptospirosis disease.

The day of diagnosis is considered the day that the first positive microbiological (e.g. serology or PCR) or histological test result was provided to the treating physician triggering systemic treatment.

In the case of post mortem diagnosis, the day of death is considered to be the day of diagnosis.

	Bilirubin	AST	ALT
day 0.	mg/dl	IU/mL	IU/mL
day 7.	mg/dl	IU/mL	IU/mL
day 14.	mg/dl	IU/mL	IU/mL
day 21.	mg/dl	IU/mL	IU/mL
day 28.	mg/dl	IU/mL	IU/mL
day 35.	mg/dl	IU/mL	IU/mL
day 42.	mg/dl	IU/mL	IU/mL

For help please contact us.

LeptoScope
Leptospirosis Registry

Go to: -- Choose Page--

Acute respiratory distress syndrome (ARDS)

You stated the patient suffered from acute respiratory distress syndrome (ARDS) during disease course of invasive Leptospirosis disease. Please provide further details on acute respiratory distress syndrome (ARDS).

Duration of acute respiratory distress syndrome (ARDS)

[ ] days

Stage of acute respiratory distress syndrome (ARDS) according to the American-European-Consensus Conference on ARDS.
- mild ARDS: PaO2/FiO2 201-300 mmHg and positive endexspiratory pressure (PEEP) > 5cmH20
- moderate ARDS: PaO2/FiO2 101-200 mmHg and positive endexspiratory pressure (PEEP) > 5 cmH20
- severe ARDS: PaO2/FiO2 < 100 mmHg and positive endexspiratory pressure (PEEP) > 5 cmH20

- ○ Mild
- ○ Moderate
- ○ Severe
- ○ Unknown
- ○ Not applicable

For help please contact us.

Back Continue

LeptoScope
Leptospirosis Registry

Go to: -- Choose Page--

Cardiac manifestation

You stated the patient suffered from a cardiac manifestation of invasive Leptospirosis disease. Please provide further details on cardiac manifestation during disease course of invasive Leptospirosis disease.

Which of the following were present?

- ☐ Supraventricular extrasystoles
- ☐ Atrial fibrillation
- ☐ Tachyarrhythmia absoluta
- ☐ Brachyarrhythmia absoluta
- ☐ Atrioventricular block type I
- ☐ Atrioventricular block type II
- ☐ Atrioventricular block type III
- ☐ Ventricular extrasystoles
- ☐ Ventricular fibrillation
- ☐ Pulseless electrical activity
- ☐ Sudden cardiac arrest
- ☐ Other
- ☐ None

Cardiac index

______ L/min

For help please contact us.

Back | Continue

LeptoScope

Leptospirosis Registry

Go to: -- Choose Page--

Neurological disorder

You stated the patient suffered from a neurological disorder due to invasive Leptospirosis infection. Please provide further details on the neurological disorder.

Which of the following were present?

- ☐ Headache
- ☐ Neck stiffness
- ☐ Paraparesis
- ☐ Tetraparesis
- ☐ Hyperreflexia
- ☐ Hyporeflexia / Areflexia
- ☐ Epileptic seizures
- ☐ Ataxia
- ☐ Altered sensorium
- ☐ Other. Please specify: ________

Which of the following neurological manifestation was present due to invasive Leptospirosis infection?

- ☐ Aseptic meningitis
- ☐ Myeloradiculopathy
- ☐ Myelopathy
- ☐ Guillain-Barré syndrome like presentation
- ☐ Meningoencephalitis
- ☐ Intracerebral bleed
- ☐ Cerebellar dysfunction
- ☐ Tremor / Rigidity
- ☐ Other. Please specify: ________

For help please contact us.

Back | Continue

LeptoScope

Leptospirosis Registry

Go to: -- Choose Page--

Ocular manifestation

You stated the patient suffered from an ocular manifestation of invasive Leptospirosis infection. Please specify the ocular manifestation.

Which of the following were present?

- ☐ Conjunctival suffusion
- ☐ Ocular muscle tenderness
- ☐ Uveitis
- ☐ Subconjunctival hemorrhage
- ☐ Chorioretinitis
- ☐ Iridocyclitis
- ☐ Papilloedema
- ☐ Papillits
- ☐ Optic neuritis
- ☐ Retinal bleed
- ☐ Cotton wool spots
- ☐ Other. Please specify: __________

For help please contact us.

Back | Continue

LeptoScope
Leptospirosis Registry

Go to: -- Choose Page--

Outcome

Was the patient alive at last contact?
- ◯ Yes
- ◯ No

Duration of hospitalization attributable to invasive Leptospirosis disease.
[ ] days

Time span between diagnosis of invasive Leptospirosis disease and last contact with the patient
If diagnosis was established post-mortem please enter "0".
[ ] days

Was the patient readmitted to hospital in the following year after invasive Leptospirosis disease attributable to invasive Leptospirosis disease?
- ◯ Yes
- ◯ No

Please state whether the patient developed chronic diseases after invasive Leptospirosis disease.
- ◯ Chronic fatigue syndrome
- ◯ Chronic cardiovascular disease. Please specify: [ ]
- ◯ Chronic pulmonary disease. Please specify: [ ]
- ◯ Chronic liver disease. Please specify: [ ]
- ◯ Rheumatic disease / Autoimmune disorder. Please specify: [ ]
- ◯ Hematological / Oncological disease. Please specify: [ ]
- ◯ Other chronic disease. Please specify: [ ]

- ☐ Please check the box if the patient developed dialysis dependent end-stage renal disease
- ☐ Please check the box if kidney transplantation was performed after invasive Leptospirosis disease

Begin of dialysis (in relation to day 0 in years)
e.g. year 5
year [ ]

Development of proteinuria
Development of protein creatinine ratio (mg/g) with regard to day of diagnosis of invasive Leptospirosis disease

	< 300 mg protein / g creatinine	300-1000 mg protein / g creatinine	1000-2000 mg protein / g creatinine	2000-3000 mg protein / g creatinine	> 3000 mg protein / g creatinine	Unknown	Not done
< 1 month							
1-6 months							
6-12 months							
1-2 years							
2-4 years							
4-6 years							
6-8 years							
8-10 years							
10 years							

For help please contact us.

Back | Continue

LeptoScope
Leptospirosis Registry

Go to: -- Choose Page--

Development of kidney function

Please provide lowest creatinine measurements within the given time-spans.

	Creatinine
month 2.	mg/dL
month 3.	mg/dL
month 4.	mg/dL
month 5.	mg/dL
month 6.	mg/dL
month 7.	mg/dL
month 8.	mg/dL
month 9.	mg/dL
month 10.	mg/dL
month 11.	mg/dL
month 12.	mg/dL
year 2.	mg/dL

year 3. [ ] mg/dL

year 4. [ ] mg/dL

year 5. [ ] mg/dL

year 6. [ ] mg/dL

year 7. [ ] mg/dL

year 8. [ ] mg/dL

year 9. [ ] mg/dL

year 10. [ ] mg/dL

Please check if no further measurements were performed

○

For help please contact us.

Back Continue

(Continued from page 33)

LeptoScope
Leptospirosis Registry

Go to: -- Choose Page--

Additional Information

If you wish to provide additional information on your case please use the space provided below.
Please be reminded that data should be anonymized to protect the patients identity.

For help please contact us.

Back Continue

LeptoScope
Leptospirosis Registry

Go to: -- Choose Page--

Thank you very much for participating in *LeptoScope*!

If you have further questions or suggestions please send an email to felix.koehler@uk-koeln.de.

For help please contact us.

Back Continue
